# Lessons Learned From Nonsignificant Findings in Exercise With Individuals With Dementia

**DOI:** 10.1093/geroni/igaa057.3119

**Published:** 2020-12-16

**Authors:** Nicole Dawson, Heather Menne

**Affiliations:** 1 University of Central Florida, Orlando, Florida, United States; 2 RTI International, Washington, District of Columbia, United States

## Abstract

The National Institute on Aging recognizes the importance of identifying promising non-pharmacological interventions (NPI) to promote health in individuals with Alzheimer’s disease and related dementias. Several systematic reviews have been completed investigating exercise in this population resulting in mixed evidence regarding efficacy across functional domains. It is critical to investigate the methodological factors from the original interventions for a true understanding of these findings as to not outright dismiss exercise as beneficial. One example is Ohio’s replication of Reducing Disability in Alzheimer’s Disease (n=508), which resulted in no significant improvements in physical performance for individuals with dementia ((gait speed (p=.81), balance (p=.82), functional reach (p=.58)). In this investigation, along with many others, researchers were not guided by key principles of exercise science leading to critical intervention design and methodological flaws. Thus, exercise interventions for individuals with dementia need to include interpretations of non-findings and report key factors affecting the outcomes.

